# A Pseudo-Value Approach to Analyze the Semantic Similarity of the Speech of Children With and Without Autism Spectrum Disorder

**DOI:** 10.3389/fpsyg.2021.668344

**Published:** 2021-07-21

**Authors:** Joel R. Adams, Alexandra C. Salem, Heather MacFarlane, Rosemary Ingham, Steven D. Bedrick, Eric Fombonne, Jill K. Dolata, Alison Presmanes Hill, Jan van Santen

**Affiliations:** ^1^Center for Spoken Language Understanding, Oregon Health & Science University, Portland, OR, United States; ^2^Department of Psychiatry, Oregon Health & Science University, Portland, OR, United States; ^3^Institute on Development and Disability, Oregon Health & Science University, Portland, OR, United States; ^4^BioSpeech Inc., Portland, OR, United States

**Keywords:** autism, language disorder, semantics, natural language proceeding, child, statistical methods

## Abstract

Conversational impairments are well known among people with autism spectrum disorder (ASD), but their measurement requires time-consuming manual annotation of language samples. Natural language processing (NLP) has shown promise in identifying semantic difficulties when compared to clinician-annotated reference transcripts. Our goal was to develop a novel measure of lexico-semantic similarity – based on recent work in natural language processing (NLP) and recent applications of pseudo-value analysis – which could be applied to transcripts of children’s conversational language, without recourse to some ground-truth reference document. We hypothesized that: (a) semantic coherence, as measured by this method, would discriminate between children with and without ASD and (b) more variability would be found in the group with ASD. We used data from 70 4- to 8-year-old males with ASD (*N* = 38) or typically developing (TD; *N* = 32) enrolled in a language study. Participants were administered a battery of standardized diagnostic tests, including the Autism Diagnostic Observation Schedule (ADOS). ADOS was recorded and transcribed, and we analyzed children’s language output during the conversation/interview ADOS tasks. Transcripts were converted to vectors *via* a word2vec model trained on the Google News Corpus. Pairwise similarity across all subjects and a sample grand mean were calculated. Using a leave-one-out algorithm, a pseudo-value, detailed below, representing each subject’s contribution to the grand mean was generated. Means of pseudo-values were compared between the two groups. Analyses were co-varied for nonverbal IQ, mean length of utterance, and number of distinct word roots (NDR). Statistically significant differences were observed in means of pseudo-values between TD and ASD groups (*p =* 0.007). TD subjects had higher pseudo-value scores suggesting that similarity scores of TD subjects were more similar to the overall group mean. Variance of pseudo-values was greater in the ASD group. Nonverbal IQ, mean length of utterance, or NDR did not account for between group differences. The findings suggest that our pseudo-value-based method can be effectively used to identify specific semantic difficulties that characterize children with ASD without requiring a reference transcript.

## Introduction

Autism spectrum disorder (ASD) is a neurodevelopmental disorder characterized by deficits in social communication and social interaction, and patterns of restricted or repetitive behavior. While atypical language use, repetitive speech, and perseverative interests are all features of the disorder as characterized by the DSM-5 (American Psychiatric Association, 2013), *quantifying* what is atypical about the language of subjects with ASD is challenging.

The use of unusual words has been found to be more prevalent in speakers with ASD (Volden and Lord, 1991); however, coding what is unusual about these words requires linguistic and clinical expertise. Additionally, identifying repetitive, stereotyped, or non-contingent speech in dialogues can be complicated by the presence of a perseverative interest. Current methods in assessing pragmatic speech are time-intensive and potentially subjective (Adams, 2002; Klusek et al., 2014). Computational methods of measuring differences in language production in subjects with ASD, such as the one proposed in this work, have the potential to provide objective, quantitative measures, which could be in turn used in clinical applications, such as evaluating response to intervention.

While there are a number of conversational impairments that we could consider, in this study, we explore what children talk about when presented with similar conversational contexts, and if that semantic content differs between children with and without ASD. We expect to capture lexico-semantic differences between diagnostic groups – e.g., differences in word use and selection between the diagnostic groups, as a measure of pragmatic speech deficits in topic maintenance. We hypothesize that when subjects are asked questions about a series of topics, that there should be some degree of similarity due to a constrained lexicon of topic-appropriate responses. In line with generally greater variability of clinical variables (language, cognitive, and behavioral) that describe the autism phenotype, we also expected that there should be a fair amount of lexical variability in those responses and that this should increase in subjects with ASD.

Prior research (Losh and Gordon, 2014; Goodkind et al., 2018) has shown that several Natural language processing semantic measures are sensitive enough to distinguish differences in word use between groups of TD subjects and those with ASD. However, as discussed below, those experiments have traditionally relied on a reference text or transcript representing an idealized typical response. This dependence can either limit the type of language samples we can evaluate, or require a poorly defined selection of “most typical transcript representative.”

We will show that these experiments, coupled with clustering and other reference document-free approaches to evaluating group differences, reveal a data set where the difference between diagnostic groups is marked not by a difference in central tendency, but rather a greater degree of variability in the language of the ASD subjects.

We propose an automated measure of “lexico-semantic similarity (LSS)” that measures across-subject similarities or divergences in an individual’s speech sample, in terms of topics discussed. This is a novel approach based on an analysis of pseudo-values (PVs) similar to that used in risk analysis (Klein and Andersen, 2005; Ahn and Mendolia, 2014). This provides us with a statistically tractable measure that can detect the sort of systematic language differences found by Losh and Gordon and Goodkind et al. and other, but *without* the requirement of a reference transcript.

Natural language processing is the subfield of computer science focused on analysis of speech and language. Many NLP tasks involve the development of computational models of semantics (i.e., what a given language sample is “about”). Such semantic models can take many forms, one of the most common being the family of vector-space models (VSMs) of semantics, which represent the meaning of a text as a vector, based on the distribution of the words that make up that text. These vectors can then be used to quantitatively analyze the semantic, or topical, content of a document or transcribed language sample.

In a simple, word-based VSM, words are represented in such a way that words with similar meanings appear close to each other in a vector space (Turney and Pantel, 2010). For example, “dog” would be measurably closer to “canine” than to “tractor.” There are a wide variety of methods for developing such models, but the simplest and most intuitive is *via* the term-context matrix: Words are organized into a matrix where column vectors represent contexts in which a word can appear (e.g., other words with which they could potentially co-occur in a document, sentence, utterance, etc., depending on the desired level of contextual granularity), and row vectors represent counts of those co-occurrences over a collection of texts or language samples. In such a model, words that appear in similar contexts (e.g., “*dogs* have four legs and a tail” and “*canines* have four legs and a tail”) are measurably more similar than those appearing in unrelated contexts (e.g., “The *tractor* tows the plow”), as the words “*dogs”* and “*canines”* occur in similar contexts and thus will have similar values along their respective row vectors. In practice, the resulting vectors are extremely high dimensional and sparse, and as such are difficult to work with. There exist a wide range of methods to produce denser and more robust representations, often by mathematically transforming a term-context matrix [as in latent semantic analysis (LSA; Deerwester et al., 1990), which uses singular value decomposition]. In recent years, new methods have been developed that use neural networks to directly estimate dense word representations without needing to first compute a full term-context matrix. One of the most common such approaches is the *word2vec* skip-gram with negative sampling model (Mikolov et al., 2013) in which a neural network is trained to use each word in a training corpus to predict its context—the words that appear around it. The resulting vector representations have been shown to capture latent relationships between words, and recent studies have used them to model meaning in a wide variety of applications (Pilehvar and Camacho-Collados, 2020).

Vector-based semantic methods have been used successfully in applications, such as document retrieval, word-sense disambiguation, and synonym identification (Turney and Pantel, 2010). Vector space semantic representations have also been used successfully to establish group differences between the language of subjects with ASD and typical development (TD) on tasks, such as semantic fluency (Prud’hommeaux et al., 2017) and narrative retelling (Losh and Gordon, 2014; Lee et al., 2018). As such, they form an excellent basis for an automated measure of the differences in what people with and without autism talk about.

Approaches to apply VSMs to the analysis of language samples, such as those referenced above, typically follow a common pattern: A subject’s language sample is transformed into a vector using one of the above-mentioned methods, as is some sort of “reference” sample. A measure of similarity between the two vectors is computed (e.g., by measuring angular distance, though many other methods exist), and then, this measure is used to represent the similarity between the two language samples, which in turn will be interpreted in whatever manner is appropriate for the task at hand.

For example, in the case of a narrative retelling task, one would use the actual text of the target narrative itself as the reference sample; a subject who was able to perform the task well (i.e., whose retelling tracked the narrative closely) would produce vectors that were much more similar to the reference vector than would a subject whose retelling was missing multiple story elements. Continuing the example, one might expect that the similarity scores thus derived from a population with impaired working memory would be, on average, lower than those from a population with intact memory. This hypothesis could be investigated using any number of statistical techniques, just as one might analyze any other quantitative metric.

A key consideration in this process is the choice of what to use as the reference sample. In the case of narrative recall tests, the obvious and valid choice is to use the ground-truth target narrative document. For other language-related assessments, however – notably including several that are clinically relevant for ASD – the question of what to use as a reference sample is less clear.

In their 2014 investigation, Losh and Gordon used LSA on a term-document matrix as a semantic representation for transcripts of narrative recall and picture book narration tasks carried out by typically developing subjects, as well as subjects with ASD (Losh and Gordon, 2014). For the narrative recall task, subject transcripts were pairwise compared in LSA space with the text of the original story. For the picture book narration task which lacked an underlying standard text to use as a language sample, the researchers compared subject transcripts to an “empirically derived standard” generated by taking the centroid of the four most centrally positioned transcripts in the LSA space, with the intuition that this represented “the center of shared meaning across the different individual narratives” (Losh and Gordon, 2014). They found statistically significant differences in the narrative recall task between the TD and ASD groups, with TD subjects being more similar to the reference text than subjects with ASD, but no difference in the picture book narration task. Losh and Gordon further suggested that the differences in results between their tasks were due to the relative complexity of the two activities.

Goodkind et al. (2018) followed a similar experiment approach, though their method of producing vector representations of their subjects’ language used the *word2vec* representations of transcripts of conversational language of subjects with and without ASD, as opposed to the narrative retelling and picture description tasks studied by Losh and Gordon. As this task lacked a natural reference document, Goodkind et al. (2018) selected several of their subjects’ transcripts to use as a reference. This reference document was chosen by clinicians from the set of TD transcripts as a “gold standard” of typically developing language. They found that average similarity scores between that reference and the remaining subjects’ transcripts differed significantly between the TD and ASD groups.

While this is a step toward an automated measure of LSS, there are limitations inherent in this methodology. First, there is little clinical foundation in how to choose a “most representative” typically developing transcript. Additionally, as we will show, we found that the degree to which their measure is sensitive to group differences in language use varies strongly by the selection of a reference transcript.

From a more theoretical standpoint, we posit that the conversational language task studied by Goodkind et al. differs from the narrative retelling and picture description tasks studied by Losh and Gordon in a way that is extremely relevant to attempts to apply automated methods to its analysis. The narrative retelling and picture description tasks are both semantically grounded in a way that a conversation is not: For the retelling task, there is a specific written narrative that the subject is meant to reproduce, and for a picture description task, the contents of the picture are intended to heavily inform the specific words and phrases that the subject produces. Even in a fairly structured conversational setting, such as that found in the ADOS, we would expect substantial between-subject lexico-semantic variation on this task, independent of diagnostic status, simply because of the open-ended nature of conversation. For this reason, we believe that relying on reference transcripts for the analysis of conversational language samples is fundamentally limiting in ways that it is not for more semantically grounded tasks.

Our goal in this work was to develop a novel methodology to allow us to use the powerful and flexible VSMs to establish language-based differences in conversational transcripts, but to do so without relying on such a reference document. To accomplish this, we drew inspiration from recent applications of pseudo-value analysis in risk assessment and survival analysis.

Pseudo-values at first glance are an intermediate step in jackknife estimation; however, they have several interesting statistical properties of their own. When treated as observations, pseudo-values can be viewed as an individual’s contribution to the estimation over the entire sample (Andersen and Pohar Perme, 2010). Tukey asserted that pseudo-values of an estimator could be treated as approximately independent and identically distributed random variables (Tukey, 1958). These properties have proven useful in risk assessment because it allows direct modeling of complex estimation on right-censored and interval-censored data (Sabathé et al., 2019). As illustrated later in this report, applying a pseudo-value approach to an estimation of overall group similarity allows us to compare the LSS of TD subjects and those with ASD in a way that is statistically tractable.

In this work, we have three objectives. *Objective 1:* to develop pseudo-value-based approaches to generate a stable, robust measure of LSS for measuring differences in language use. *Objective 2:* to demonstrate that pseudo-value approaches can be used to establish group differences between the language of children with and without ASD. We hypothesize that transcripts of typically developing subjects will be more similar to both each other and to the group overall, and that this will manifest as subjects with ASD having lower LSS scores overall than TD subjects, and more variability within the group. *Objective 3:* to leverage the statistical properties of our pseudo-value based measure and investigate trends in both the mean and dispersion of our measure with respect to other measures of language fluency and development, such as mean length of utterance in morphemes (MLU), number of distinct word roots (NDR), verbal IQ (VIQ), and performance IQ (PIQ). We expect that our findings will co-vary with, but will not be entirely accounted for by MLU, NDR, VIQ, and PIQ.

## Materials and Methods

### Participants

Participants in this study were a subset of children aged between 4 and 8 years who participated in a larger study and were recruited from various healthcare and community sources, in the Portland, OR, metropolitan area (see Hill et al., 2015 for further study details). Participating families were fully informed about study procedures and provided written consent. The Oregon Health & Science University Institutional Review Board approved all experimental procedures.

All participants were evaluated with the ADOS Module 3 (Lord et al., 1999). Due to small numbers of females and potential confounding by sex of language differences, only males were eligible for this investigation. All participants spoke English as their native and first language. Children were excluded for any of the following conditions: (1) identified metabolic, neurological, or genetic disorder; (2) gross sensory or motor impairment; (3) brain lesion; (4) orofacial abnormalities, such as cleft palate; and (5) intellectual disability. A certified speech-language pathologist confirmed the absence of speech intelligibility impairments during an initial screening. All participants scored 70 or higher for full-scale IQ on either the Wechsler Preschool and Primary Scale of Intelligence (Wechsler, 2012) if under 7 years old, or the Wechsler Intelligence Scale for Children (Wechsler, 1949) if older. Children in the TD group had to have scores below threshold on both the ADOS and the Social Communication Questionnaire (SCQ; Rutter et al., 2003) no personal history of neurodevelopment disorder, such as attention deficit hyperactive disorder, and no family history of ASD or specific language impairment.

For participants in the ASD group, best estimate clinical (BEC) consensus judgment was used to confirm the presence of ASD according to DSM-IV-TR criteria (Segal, 2010). Judgments were made by a panel of two clinical psychologists, one speech-language pathologist, and one occupational therapist, all of whom had clinical experience with ASD. BEC consensus by experienced clinicians is considered the gold standard for diagnosis of ASD (Spitzer and Siegel, 1990; Klin et al., 2000). In addition, children in the ASD group scored above threshold on both the ADOS (Lord et al., 1999) with the revised algorithm and the SCQ (Rutter et al., 2003) with the recommended research cutoff score of 12 (Lee et al., 2007). The final sample comprised 38 subjects with an ASD diagnosis and 32 TD subjects, all males.

The sample characteristics are summarized in Table 1. The subjects with ASD were slightly older than those without ASD. There was a 19-point difference in full IQ between the two groups that was accounted for by higher VIQ and PIQ in the TD group. There was no significant difference between the two groups in the language measures (MLU and NDR).

**Table 1:** Sample characteristics.

	Mean (SD)		*p*
	TD*n* = 32	ASD*n* = 38	
Age (years)	6.0 (1.2)	6.8 (1.2)	0.008
Full-scale IQ	119.1 (10.2)	100.5 (16.3)	<0.001
Performance-IQ	118.9 (13.6)	97.7 (17.7)	0.029
Verbal IQ	118.7 (11.8)	110.8 (16.8)	<0.001
MLU	4.7 (0.84)	4.3 (0.94)	0.07
NDR	449.8 (88.1)	422.3 (135.7)	0.31

MLU, mean length of utterance in morphemes; NDR, number of distinct word roots.

### Procedures

Participants completed a battery of experimental tasks and cognitive, language, and neuropsychological assessments over approximately six 2–3 h sessions. The Wechsler scale tests were administered as described above. The Wechsler scale tests were used to estimate VIQ, PIQ, and full-scale IQ.

All participants received the Autism Diagnostic Observation Schedule-Generic (ADOS; Lord et al., 1999) a semi-structured autism diagnostic observation, administered by an experienced and trained clinician. Module 3 requires fluent language from the participant and comprises 14 tasks. The ADOS recordings were manually transcribed using SALT transcription conventions by a team trained to a research level of reliability. Transcribers were unaware of the subjects’ diagnostic status. The resulting transcripts were used to calculate other measures, including MLU.

We used the ADOS as the source of our language sample. Its widespread uses as a diagnostic instrument for ASD, coupled with the focus of several of its activities on eliciting naturalistic conversational language made it ideal.

### Transcript Processing

We selected the subset of four conversation-based activities of the ADOS, due to their focus on spontaneous speech and the relatively structured nature of the conversations. Examiners in these sections ask a scripted set of questions insuring a common conversational context between participants. The conversations are designed to elicit different responses from typically developing subjects and those with ASD (Lord et al., 1999), and as such, our measure of LSS should be sensitive to the variability and group differences in the resulting conversations. Specifically, we restricted our analysis to transcripts of the “Emotions,” “Social Difficulties and Annoyance,” “Friends, Relationships and Marriage,” and “Loneliness” ADOS activities.

The resulting transcripts were analyzed *via* a series of automated text processing tasks. We first identified subject utterances within the transcript. Labeled content mazes (repetitions and revisions) and tokens annotated as sound effects or incomplete words were removed. We also chose to exclude pause fillers (such as “uh” and “um”). While recent work has shown interesting differences of usage of these terms (Gorman et al., 2016; McGregor and Hadden, 2020), we consider these to be pragmatic language features and not directly translatable to a semantic representation. All tokens were then case-folded into lower-case.

The transcripts were then converted to vector representations using the word2vec VSM. Each word in a subject’s transcript was transformed into a 300-dimensional word vector *via* the application of a word2vec model pre-trained on the Google News Corpus (Mikolov et al., 2013). This model is trained on approximately 100 billion words from the Google News corpus. While the mismatch between child speech and the Google News Corpus is a possible limitation of our study (which we discuss in Future Research section), using this model allowed us to perform a direct comparison with the results of Goodkind et al. (2018). Words that were out-of-vocabulary for the model were excluded (this resulted in the exclusion of 174 distinct terms out of a vocabulary of 4,288 words). The vast majority of these excluded terms were proper names and function words (such as “and,” “a”), which are removed from the model as they perform a syntactic rather than lexico-semantic function.

At this point, a transcript was represented as a sequence of all of the word vectors that made it up. Following the method of Goodkind et al. (2018), we then summed these word vectors to combine them into a single transcript vector, which was then normalized to unit length to control for differences in transcript length.

The similarity between each of these transcripts was then measured by the application of the cosine similarity function, which translates the angle between two vectors into a scalar value between 0 and 1 (Equation 1). This is a standard measure of similarity in a VSM, which gives a measure that is robust to differences in vector length. For any two vectors A and B, the cosine similarity is

(1) $$similarity\;\left(A,B\right)=\frac{A\cdot B}{∥A∥\;∥B∥}\;$$

This is equivalent to the cosine of the angle between vectors A and B.

### Analysis

#### Objective 1: Pseudo-Value Measure of LSS

Our first aim was to build a stable, robust measure of LSS that can identify group differences based on word use but without dependency on a manually selected reference transcript.

We started by investigating the effect of the arbitrary selection of a reference transcript. We replicated the results of Goodkind et al. on our data; however, rather than select a *single* reference transcript as per Goodkind et al., we varied the selection of reference transcript between *all* 32 TD subjects. For each selected reference transcript, we calculated the cosine similarity of all of the other subjects’ transcripts to this reference. We then compared the mean similarity scores to this reference by diagnostic group.

To further understand these results, we performed dimensionality reduction to visualize the distribution of these transcripts in the vector space. We used Kruskal’s approach to isometric multidimensional scaling (IsoMDS; Kruskal, 1964) to reduce the 300-dimensional vector space to two dimensions and plotted the results.

Our experiments confirmed the instability caused by the Goodkind et al. method’s dependency on reference transcripts (see Results). Furthermore, our IsoMDS analysis indicated that the differences between the TD and ASD groups were less a matter of differing group centroids than it was of *increased variability* in the ASD transcripts (see Discussion). We therefore pursued an analytical approach based on pseudo-values.

Pseudo-values (PVs) provide us a single scalar value for each subject, measuring that subject’s statistical leverage on the overall similarity of our data set. These PVs are also statistically independent and can, as such, be used in traditional statistical analyses. This allowed us to pursue Objectives 2 and 3 in a straightforward manner – evaluating group differences, and exploring trends in the mean and dispersion of our measure with respect to other measures of language fluency and development.

Our approach to computing PVs was as follows. We first calculated the pairwise similarity of the transcripts between each of the subjects by measuring the cosine similarity between the vector space semantic representations of each transcript. As these similarity values were heavily skewed toward 1.0, we applied the Fisher Z transformation to all of the similarity scores.

Following the jackknife methodology as described by Miller (1974), we generated a relevant pseudo-value representation, by first considering an estimator for the mean pairwise similarity given our entire set of subjects. With sample Xmade up of n observations, and some estimator ϕ, we can define a pseudo-value $p_{i}$ for each subject *i*, as follows:

(2) $$p_{i}\left(X\right)=n\phi \left(X\right)-\left(n-1\right)\phi \left(X_{\neg i}\right)$$

where $X_{\neg i}$ is the original sample with the *i*th observation removed. In our case, ϕis the mean pairwise similarity of all transcripts it is applied to. As such, $\phi \left(X_{\neg i}\right)$ is the leave-one-out estimate of mean pairwise similarity, including only pairs that do not include the transcript of subject i. In other words, we subtract the weighted leave-one-out estimate from the weighted overall mean, resulting in a number representing the unique contribution of a given subject to the overall mean. We then used these pseudo-values as our measure of LSS in subsequent analyses.

#### Objective 2: Establishing Group Difference

Having thus defined a measure of LSS, Objective 2 was to demonstrate that this measure could be used to establish group differences between the language of children with and without ASD. We computed LSS for each child and compared the mean LSS values between the two diagnostic groups using the Wilcoxon rank sum test to determine the statistical significance of the difference in median values.

#### Objective 3: LSS and Clinical Features

Finally, we considered whether other measures of language and development were interacting covariates with regard to our LSS measure. We used multiple linear regression with our LSS measure as the dependent (response) variable and a variety of additional measures as independent (explanatory) variables. These included performance and VIQ, as well as the language measures of MLU and NDR.

Mean length of utterance in morphemes is a common measure of a child’s overall language development (Brown, 1973; Parker and Brorson, 2016), and we used NDR as a rough measure of a child’s expressive vocabulary. Both of these measures were calculated from the conversation activities of subjects’ transcripts automatically *via* application of the tool AutoSALT (Gorman et al., 2015).

Our modeling strategy was as follows. We first created an omnibus linear regression model to predict LSS using the *lm* function in R, with main effects of diagnosis, age, PIQ, VIQ, MLU, and NDR, as well as the interactions between diagnosis and each of PIQ, VIQ, MLU, and NDR. We then simplified the model by removing all nonsignificant interaction terms at the threshold of *p =* 0.05. Our final set of model parameters can be found in Results.

## Results

### Objective 1: Pseudo-Value Measure of LSS

**Figure 1:**
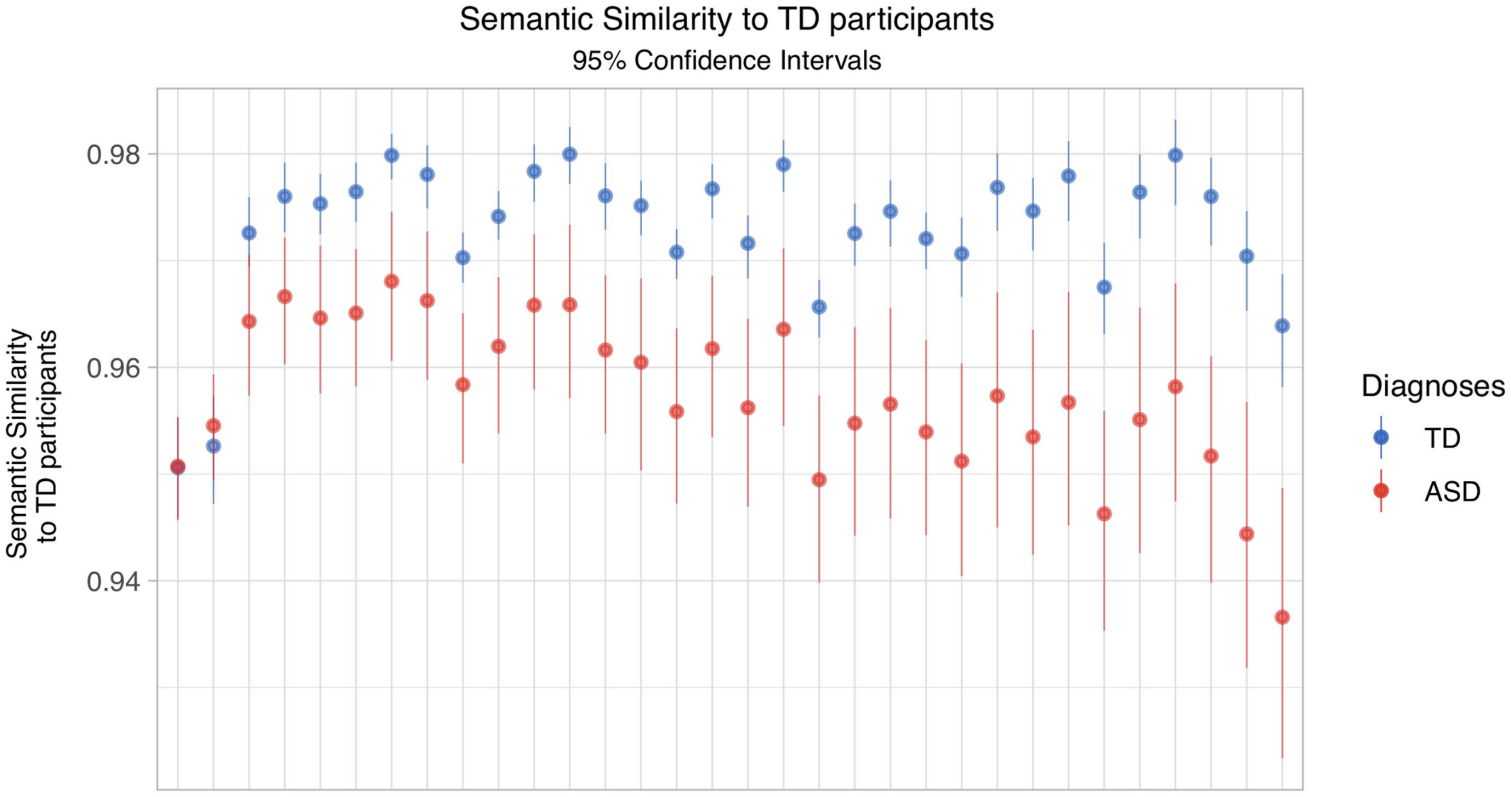
Semantic similarity by diagnostic group, varying the TD reference transcript.

When we varied the reference transcript across the set of all TD subjects, the mean similarity to that transcript was higher for the group of TD subjects than ASD subjects, for all but one reference transcript (Figure 1). We further note that the *variability* of that similarity is consistently higher for the ASD group. Crucially, the amount of variability in the reference transcripts resulted in the groups not being differentiable in 6 times out of 32.

The starkest effect of reference transcript selection can be seen in the difference between the leftmost and rightmost reference transcripts in Figure 1. For the first transcript, the means are almost identical, while in the other, there is substantial separation between the two groups.

**Figure 2:**
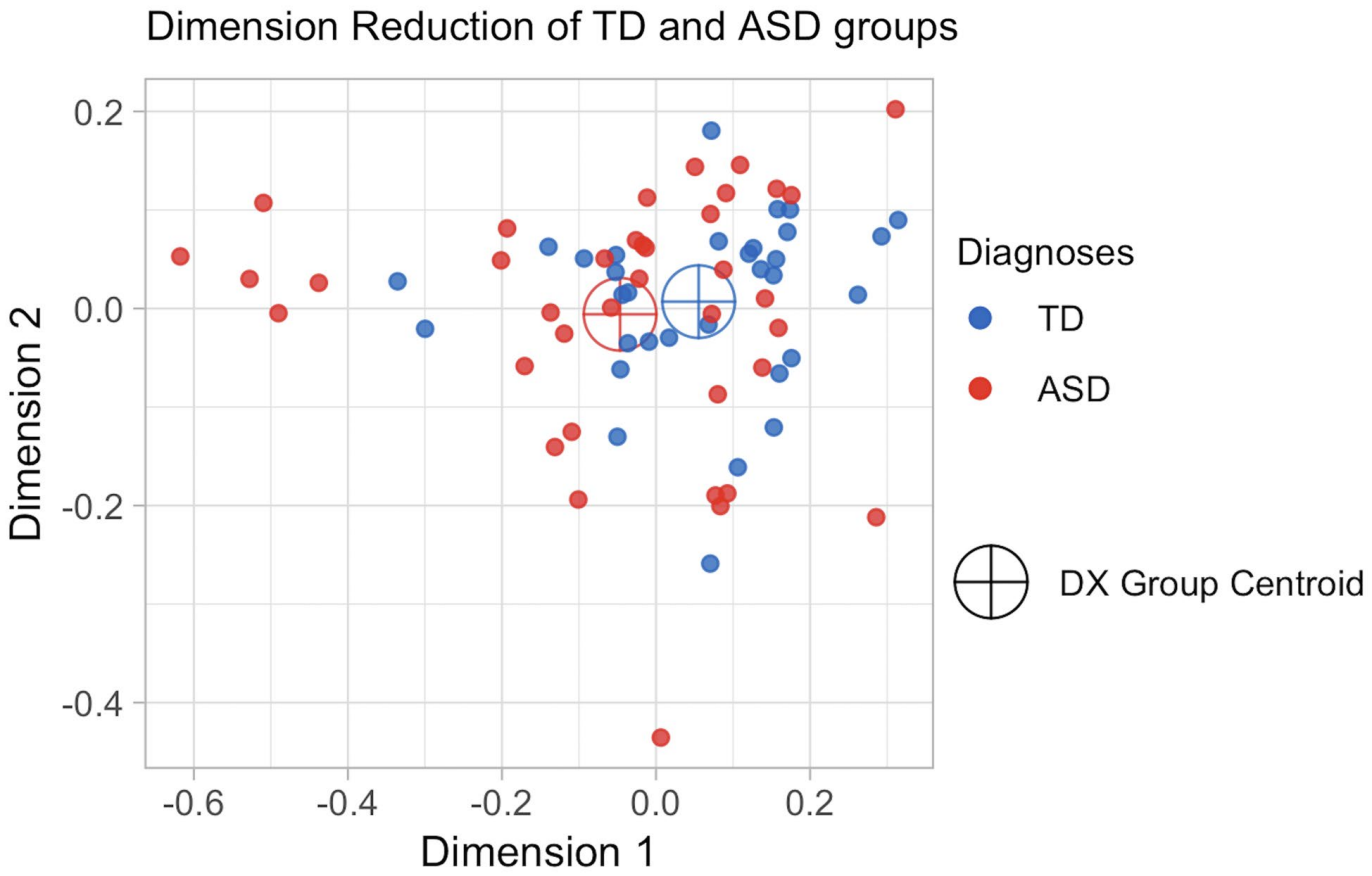
Dimensionality-reduced visualization of transcript vector space.

With the vector-space representation scaled down to two dimensions in Figure 2, we can see that the two groups have similar centroids. While there is variability in both groups, the TD subjects are more tightly clustered in semantic space, and we see more variability and outliers in the ASD group. This is borne out in the measure of total variance, a common measure of the variability of multivariate data (Rencher, 2003). The total variance of the TD group is 0.027, while the variance of the ASD group is 0.0523.

### Objective 2: Establishing Group Difference

**Figure 3:**
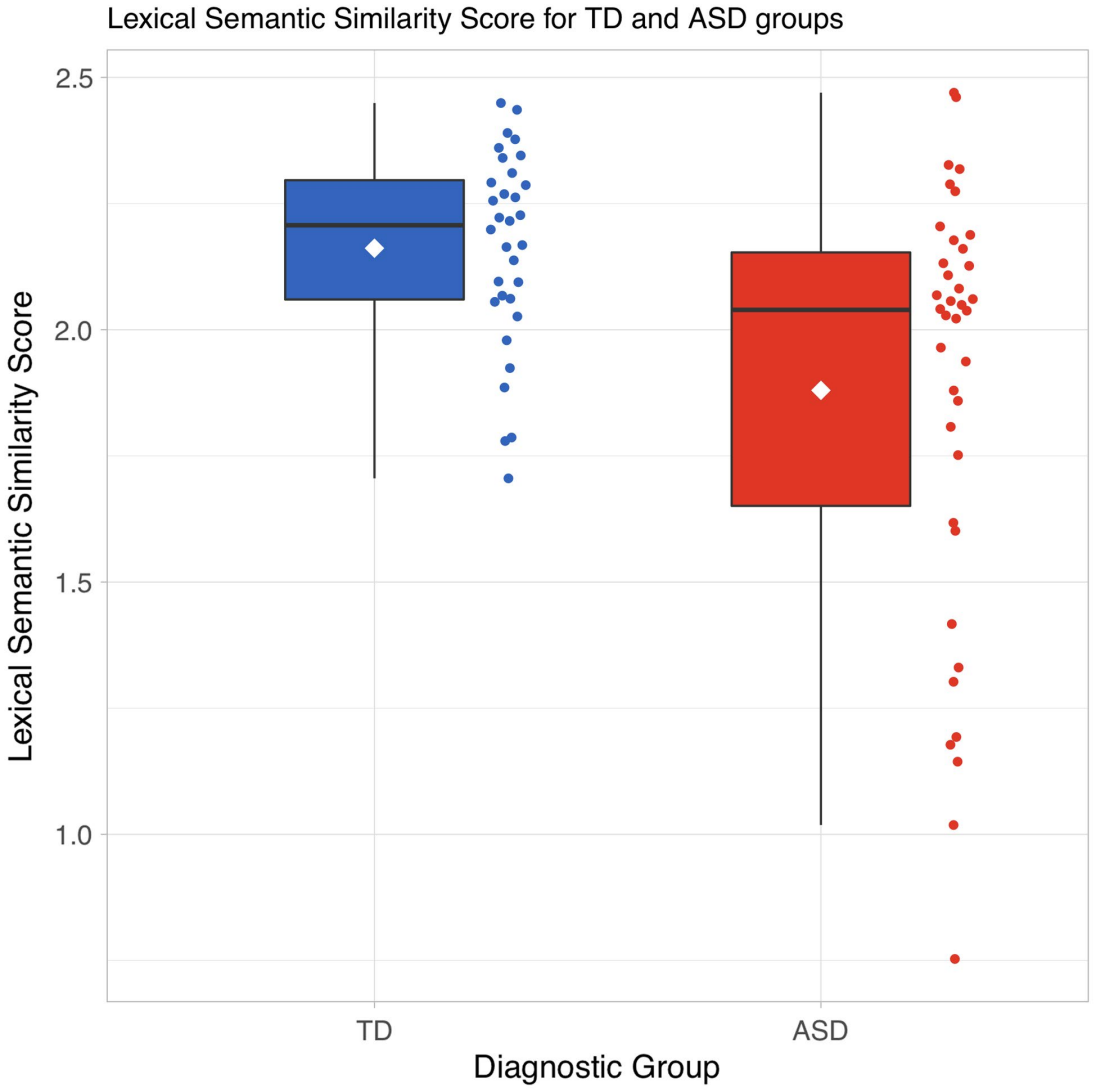
Distribution of lexico-semantic similarity scores by diagnostic group.

As shown in Figure 3, the distribution of our pseudo-value-based LSS measure for children with ASD is shifted significantly lower than the distribution for TD children (Wilcoxon rank sum test: *p =* 0.016).

The pseudo-values represent the relative leverage of individual subjects’ positions in lexico-semantic vector space. Higher values mean more similarity to the overall group. Lower values represent subjects who are relative outliers in the vector space. These should represent subjects whose language use is different from that of the rest of the group.

It is noteworthy that – as seen in the swarm plot in Figure 3 – the ASD group includes eight subjects with pseudo-value scores lower than 1.5, lower than any pseudo-value scores observed in the TD group. We will address these subjects more closely in the Discussion section.

### Objective 3: LSS and Clinical Features

After removing the nonsignificant interaction terms from our omnibus model, we were left with the final model with coefficients listed in Table 2. Even when correcting for age, VIQ, PIQ, MLU, and NDR, diagnosis is a statistically significant predictor of LSS.

**Table 2:** Regression coefficients.

Estimate	Std.	Error	*t*	value	Pr(>|t|)
(Intercept)	1.2429	0.4491	2.768	0.0074	^*^
Diagnosis	0.6929	0.3117	−2.224	0.0298	^*^
Age	0.0002	0.0027	0.082	0.9347	
VIQ	0.0044	0.0025	1.767	0.0821	
PIQ	0.0006	0.0024	0.254	0.8002	
MLU	0.0008	0.0573	−0.014	0.9892	
NDR	0.0007	0.0007	0.973	0.3342	
Diagnosis:NDR	0.0012	0.0007	1.876	0.0654	

* *p* < 0.05.

**Figure 4:**
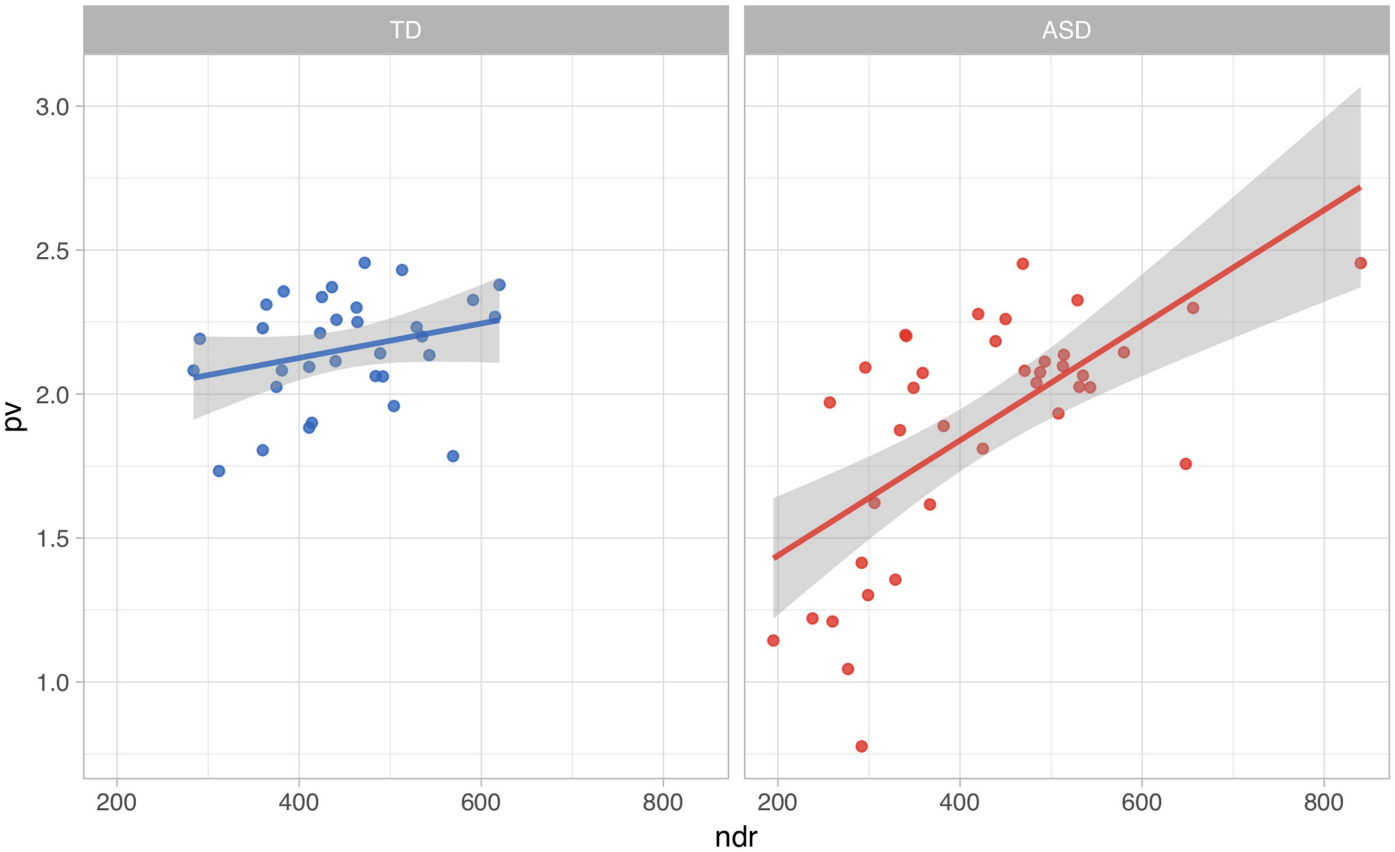
Relationship between number of distinct word roots (NDR) and lexico-semantic similarity by diagnostic group.

In the omnibus model, NDR was the only significant interaction term. Without the inclusion of this interaction, diagnosis is not significantly predictive. As we can see in Figure 4, the NDR increases, so does the LSS score.

## Discussion

In this paper, we proposed a novel application of pseudo-values to create a stable measure for analyzing LSS in conversational language between groups, which can be measured on conversation samples without additional human annotation. We believe that this is the first time pseudo-value analysis has been applied in this way.

### Objective 1: Pseudo-Value Measure of LSS

We were able to recreate the findings of Goodkind et al. (2018) on our data set. Generally speaking, we found that for all but one of the selected reference transcripts, the mean similarity was higher for children with TD and that the variability in those scores was higher for children with ASD diagnoses. We conclude that comparing vector representations of subject transcripts to a reference transcript *can* show group differences in mean value, as per Goodkind et al. However, due to variability in the TD transcripts, the size and significance of the results are heavily dependent on the selection of reference transcript.

This is not surprising. While the ASD group was chosen due to a clinical assessment of ASD, the non-ASD group selection criteria were simply the lack of an ASD diagnosis. Even if solely measured by VIQ, we find quite a bit of variation in the language ability of the non-ASD group. It is possible that the variability introduced by changing reference transcript would be increased in a more naturalistic sample, as it is likely that this data set is *more* homogeneous than the actual population due to selection bias. It was satisfying to see that the method of Goodkind et al. generalizes to our data set, but as a repeatable metric it proved to be too dependent on the selection of a reference subject for our needs.

The results in Figure 2 were suggestive. In such a plot, if children with ASD systematically spoke about a topic that was different from that of children without ASD (e.g., if all children with ASD used words about “school” and all children without ASD used words about “dinosaurs”), we would see a distinct cluster for each of the two diagnostic groups. Instead, we see that the transcripts of the two groups cluster around similar centroids, with the subjects without ASD grouped a bit more tightly in the middle and with more variability in the subjects with ASD. This motivated our use of pseudo-values to consider a given subject’s difference from the overall mean rather than the group centroids.

This pattern of similar centroids but increased variability in the ASD group is consistent with the findings of Losh and Gordon (2014) on narrative retellings. One possible avenue that could allow an empirical selection of a reference document would be to follow the intuition of Losh and Gordon and choose the most centrally located transcripts. However, this would skew any statistical analysis we would want to do of possible covariates by removing the most (at least theoretically) representative TD subjects from consideration.

### Objective 2: Establishing Group Difference

Moving to a pseudo-value-based measure of LSS gives us a measure that is statistically tractable without a dependency on a single reference transcript. LSS does, in fact, distinguish between ASD and non-ASD groups. Subjects in the non-ASD diagnosis group have higher scores in aggregate, suggesting that the semantic choices of subjects without ASD are more similar to that of the overall group. ASD subjects have a lower value on our measure suggesting more examples of outliers in the same space.

The difference in diagnostic groups seems to be largely due to the group of subjects with LSS scores lower than 1.5. While investigating these patterns, we found them to be the same subjects with ASD that can be seen in Figure 2 at the edges of the dimensionality-reduced view of the vector space (i.e., the cluster of points in the lower-right-hand quadrant).

This is perhaps unsurprising, as the IsoMDS algorithm preserves the relative ordering of pairwise distance, and our LSS measure is a measure of leverage calculated over aggregated pairwise distances. It does seem appropriate, however, that the seeming outliers in our lexico-semantic vector space would have the lowest scores in our measure.

### Objective 3: LSS and Clinical Features

The results documented in Table 2 show that diagnosis is a statistically significant predictor of LSS even when controlling for possible covariates and other measures of language fluency (VIQ, MLU, and NDR). In fact, it is the *only* significant predictor. This suggests that we are, indeed, measuring something different in the language use of subjects with and without ASD diagnoses.

It is important to consider the effect of the interaction of diagnosis with NDR. The trend of LSS to increase with NDR is much more pronounced in the subjects with ASD, where a larger productive vocabulary seems to correspond to language more similar to that of the overall group. This interaction seems to be heavily impacted by the same eight subjects with low LSS and ASD discussed identified in the results for Objective 2. While both the TD and ASD groups have subjects with relatively low NDR scores (<300), only the ASD group has a subset of those with LSS scores below 1.5.

The finding that LSS is not fully explained by number of distinct roots is particularly encouraging, as NDR is the most explicitly lexical of our external language measures. This suggests that the LSS difference cannot simply be dismissed as an artifact of vocabulary size.

### Future Research

While word2vec has been used successfully in tasks related to semantic term and document similarity, it remains a possible concern that our approach to generating document representations by using the linear compositionality property of word2vec vectors (summing word vectors to generate the document vectors) is not the best way to capture document meaning. An investigation into the correlation of such models to human judgment of semantic similarity (“about-ness”) would be welcome.

Additionally, there is work to be done on improving the quality of the vector representations of the words themselves. We used word vectors trained on a news corpus. While there are few large corpora of spoken language for children in the age range studied here, model adaptation or using a conversation-based training corpus could result in better representations of child conversational speech. Additionally, the importance of the eight subjects with low LSS in the group differences opens a number of interesting areas for research. The fact that the low LSS subjects all have low NDR scores caused us to look at the other language measures for these subjects. These subjects are all in the lower 50% of MLU as well.

As both MLU and NDR are highly correlated with the length of the resulting transcript, it is important to consider the effect of document-length normalization methods on our LSS measure. Our current approach (reducing the document representation to unit length) preserves the cosine similarity of un-normalized document representations. However, there are other approaches to create comparable document models, such as weighted averages of the word vectors composing a document, or more recent document models, such as doc2vec (Le and Mikolov, 2014), Word Movers Embedding (Wu et al., 2018), or BERT (Devlin et al., 2019); future work will explore ways that these more advanced models could be used in this setting.

### Conclusion

We found statistically significant group differences in language use between ASD and TD subjects. Additionally, we found that this result was not fully explained by standard measures of language ability. The authors consider this to be the first part of a chain of analytical tools for quantifying issues with conversational speech.

Significantly, we propose a method of calculating LSS that is independent of any single reference transcript. This approach of utilizing pseudo-values to represent a subject’s leverage on the sample set has potential application in any analysis of group differences that involves the computation of pairwise similarity scores, including any vector semantic document representation.

The findings suggest that NLP methods can be effectively used to identify specific instances of some of the conversational difficulties that characterize children with ASD. Computational methods of measuring differences in language production in subjects with ASD, such as the one proposed here, have the potential to provide objective, quantitative measures, which could be useful in clinical applications, such as response to intervention.

## Data Availability Statement

The raw data supporting the conclusions of this article will be made available by the authors, without undue reservation.

## Ethics Statement

The studies involving human participants were reviewed and approved by the Oregon Health & Science University Institutional Review Board. Written informed consent to participate in this study was provided by the participants’ legal guardian/next of kin.

## Author Contributions

JA, AH, JS, and SB contributed to the experiment design. AS created the figures. JA wrote the first draft of the manuscript. RI wrote a section of the manuscript. JK and EF provided clinical insight. All authors contributed to manuscript review and revision.

### Conflict of Interest

JS was employed by company BioSpeech Inc. The remaining authors declare that the research was conducted in the absence of any commercial or financial relationships that could be construed as a potential conflict of interest.
